# Adenoid (Acantholytic) Squamous Cell Carcinoma of Mandibular Gingiva

**DOI:** 10.1155/2021/5570092

**Published:** 2021-07-22

**Authors:** T. Raut, S. Keshwar, M. R. Jaisani, A. Shrestha

**Affiliations:** ^1^Department of Oral Pathology, College of Dental Surgery, BP Koirala Institute of Health Sciences, Dharan, Nepal; ^2^Department of Oral and Maxillofacial Surgery, College of Dental Surgery, BP Koirala Institute of Health Sciences, Dharan, Nepal

## Abstract

**Introduction:**

Adenoid (acantholytic) squamous cell carcinoma (ASCC) is a histological variant of squamous cell carcinoma which occurs mainly in the sun-exposed areas of the head and neck region. It is commonly seen among males which mainly occurs in the sixth and seventh decade of life with lip being predominately affected. Limited scientific literature is documenting the intraoral presentation of ASCC in contrast to its usual extraoral lesions associated with the skin. Characteristic pseudo glandular alveolar space formation seen in ASCC often mimics carcinoma of salivary gland origin. In-depth knowledge of histopathological features of ASCC is important to diagnose this uncommon variant. *Case Description*. An 80-year-old female presented with the chief complaint of the nonhealing lesion in the right lower back region of the jaw for 2 months, associated with pain. A provisional diagnosis of oral cancer was considered, and an incisional biopsy was done. Histopathological presentation of the epithelial tumor island, pseudo glandular duct-like structures, and neoplastic cells showing features of dysplasia and keratin pearl formation confirmed the diagnosis as adenoid (acantholytic) squamous cell carcinoma.

**Conclusion:**

The histopathological presentation of adenoid (acantholytic) squamous cell carcinoma reflects the prognosis and metastatic behavior of the diseases. The knowledge of histopathological features of ASCC would be a guide to the untrained eye for the diagnosis and management of this uncommon variant to minimize the rate of metastasis or reoccurrence.

## 1. Introduction

Squamous cell carcinoma (SCC) is the most common cancer of the head and neck region. It follows a geographical pattern of incidence which is highest among South Asians [1]. Squamous cell carcinoma has several histopathological variants such as verrucous, spindle, basaloid, adenosquamous, adenoid, and undifferentiated type [2].

The behavioral pattern and prognosis of SCC often can be correlated with molecular analysis for aneuploidy, loss of the tumor suppressor gene, and loss of heterozygosity. However, few histopathological variants of SCC guide the prognosis and biological behavior on its routine microscopic presentation; basaloid and adenoid variants are two among them [3, 4]. Adenoid (acantholytic) squamous cell carcinoma represents 2-4% of SCC which affects mainly the sun-exposed areas of the skin [5]. Goldman et al. in 1977 reported the first case of oral cavity-associated adenoid (acantholytic) squamous cell carcinoma (ASCC). Here, we present a case of a female patient diagnosed with ASCC. This case report is intended to highlight the rare pathology familiarizing its histopathological features in regards to the biological behavior.

## 2. Case Report

An 80-year-old female presented with a complaint of a nonhealing lesion in the right posterior region of the lower jaw for two months. The lesion was gradually increasing in size associated with moderate, intermediate-type pain with purulent discharge. She had a habit of chewing tobacco with slaked lime for 60 years, 6-7 times per day which she kept for 10-20 minutes at variable sites in the vestibular region and spit. She also had a habit of smoking tobacco for 50 years, 5-6 cigarettes per day, and had no other significant medical history.

**Figure 1:**
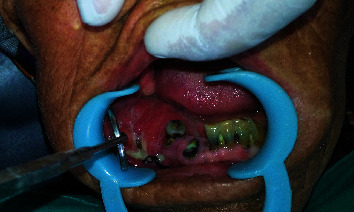
Erythematous ulcerative growth involving the right alveolar ridge and vestibule.

On extraoral examination, there was a localized swelling around the body of the mandible on the right side with a single, firm, mobile, and tender lymph node approximately measuring 1 × 1.5 cm^2^ in maximum dimension palpable in the submandibular region. Intraoral examination revealed a single, soft, erythematous swelling with ulceration extending from 44 to 48 involving the vestibule, buccal mucosa, edentulous alveolar ridge, and lingual mucosa extending towards the floor of the mouth, measuring approximately 2.5 × 2.5 cm^2^ in maximum dimension. The surface of the lesion shows localized thick purulent discharge and is tender on palpation with peripheral induration. There was a root stump associated with 42-44 and carious exposure on 33-41 with edematous gingiva (Figure 1). The panoramic radiograph revealed a homogenous radiolucent lytic lesion involving the body and angle of the mandible on the right side with irregular cortical margin in the body area extending from 44 to 46.

**Figure 2:**
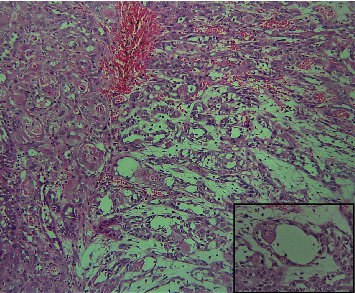
Section showing epithelial tumor islands with keratin pearls and pseudo glandular duct-like structures (H&E, 10x); inset: pseudo glandular duct-like structures lined by a single layer of cells with the dyskeratotic cell within the space (H&E, 40x).

**Figure 3:**
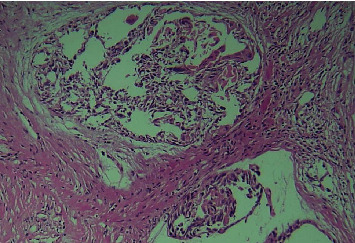
Epithelial tumor islands within connective tissue showing acantholysis and loss of cellular attachment (H&E, 10x).

**Figure 4:**
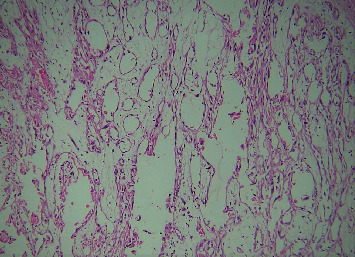
Space giving the pseudo glandular presentation (H&E, 10x).

The clinical diagnosis of oral carcinoma was made, and an incisional biopsy was suggested. A single, soft tissue measuring 13 × 7 × 5 mm in maximum dimension, creamish white in color, and firm in consistency was obtained and fixed in 10% neutral buffered formalin. The fixed tissue was processed, embedded, and sectioned with the soft tissue microtome at a thickness of 3 *μ*m and stained with routine hematoxylin and eosin stain. The histopathological section revealed epithelial tumor islands within connective tissue showing the central area of acantholysis. Islands show features of dysplasia in the form of dyskeratosis, mitotic figures, cellular and nuclear pleomorphism, and keratin pearl formation (Figures 2 and 3). Numerous pseudo glandular structures lined by a single layer of flat to polygonal cells were appreciated (Figure 4). Periodic acid-Schiff stain confirmed these structures to be nonglandular without any mucinous components. The lumen shows detached keratinocytes with the intense eosinophilic cytoplasm (Figure 2). Surrounding connective tissue was fibrocellular with inflammatory cell infiltrates. No lymphovascular or perineural invasion was evident. Reviewing the diagnostic criteria, a final diagnosis of adenoid (acantholytic) squamous cell carcinoma was made. The patient was suggested for radiotherapy at the cancer center because of the extensive nature of the lesion and age. However, the patient refused to visit any cancer center and passed away four months following the diagnosis.

## 3. Discussion

Adenoid (acantholytic) squamous cell carcinoma (ASCC), first described by Lever in 1947, is now a well-recognized histopathological variant of squamous cell carcinoma that rarely occurs within the oral cavity [6, 7]. The condition is prevalent in sun-exposed areas of the head and neck region where radiation is claimed to be the etiological agent whereas the intraoral presentation lacks this etiological basis. Sun exposure has also been associated with increased incidence of ASCC in the lower lip [8]. In contrast to conventional OSCC where alcohol and tobacco are the prime etiological agents, no such definitive etiology has been ascribed to ASCC. In the case report of Bhogavaram et al., trauma has been emphasized as the origin of ASCC; however, due to the paucity of its occurrence, etiopathological basis has not been well identified [9]. O'Shea et al. in their immunofluorescence-based study summarized that the loss of cellular adhesion in ASCC is primarily due to desmosomal defects specifically desmogleins 1 and 2 or desmoplakin or both [10].

Chandrakala et al. in 2018 have summarized a total of 45 cases of ASCC in the oral cavity reported in English language literature to date making this pathology uncommon. The literature shows male predilection and lip to be the predominant site (22 cases) of occurrence which includes 16 cases of the lower lip, 4 cases of the upper lip, and the remaining 2 cases which were not specified. Cases other than the lip were found in the gingiva, tongue, floor of the mouth, and buccal mucosa [5]. After an extensive literature review in year 2018 [5], seven more cases have been added to the literature which includes lesion associated with the upper lip, maxilla, buccal mucosa, and tongue region [8, 9, 11, 12]. The present case is seen within the mandibular jaw. A thorough literature review on the cases of the mandibular jaw revealed a total of 10 published cases to the best of our knowledge (Table 1).

Lasser et al. and Leon et al. summarized the diagnostic criteria for ASCC as follows: (i) the basic cell should be keratinizing squamous type, (ii) adenoid structure consists of a rounded space with a definitive wall, principally of one cell thickness, and (iii) the lumen of the adenoid/ductal structure contains single or group of dyskeratotic acantholytic cells [7]. Based on microscopic features, ASCC has many synonyms such as adenoid SCC, pseudo glandular SCC, SCC with gland-like features, angiosarcoma-like SCC, pseudo vascular adenoid SCC, and epithelioma dyskeratoticum segregans [13, 14]. The combined microscopic features of the duct-like structures with conventional SCC often give a list of histopathological differential diagnosis which includes conventional squamous cell carcinoma, adenosquamous cell carcinoma, mucoepidermoid carcinoma, and angiosarcoma. Conventional SCC lacks duct-like structures lined by a single layer of polygonal cells. Adenosquamous cell carcinoma has both the component similar to ASCC but stains positive for periodic acid-Schiff (PAS), Alcian blue, and mucicarmine. Mucoepidermoid has the characteristic presence of three different types of cells such as mucous, epidermoid, and intermediate type with the positivity of PAS, Alcian blue, and mucicarmine. Angiosarcoma also shows close histopathological resemblance to ASCC where the sections have to be thoroughly evaluated for the presence of dysplastic epithelial tumor islands with keratin pearl formations. Vascular markers, CD31 and CD34 along with Fli-1 protein, would be diagnostic for angiosarcoma [2, 5, 13].

Skin-associated ASCC has poor prognosis [14]; similarly, ASCC of the larynx is also commented for its aggressive behavior showing regional lymphadenopathy and reoccurrence [15, 16]. The biological behavior of ASCC of the oral cavity is considered to have poor prognosis by some author to variable by other which might be due to limited reported cases [2, 5, 6, 13]. Detachment of neoplastic cells due to acantholysis is correlated with its distant metastasizing behavior [10]. Ishikawa et al. in their study found that 46-month survival for ASCC was zero percent whereas 30-month survival was just 20.4% and concluded the ASCC of the oral cavity to have an extremely poor prognosis. The study has also analyzed the treatment protocol opted in various ASCC patients. Out of 13 cases evaluated, 7 were treated with surgery (alone and with radical dissection), in which 6 showed a good postoperative course without evidence of diseases whereas 6 cases were treated with adjuvant (chemotherapy or radiotherapy) along with surgery, all of them showed reoccurrence and died due to diseases within 7-46 months of primary surgery showing poor prognosis. Despite limited cases and a short period of follow-up, the study suggested that radical surgery might be very important in the patient of oral ASCC.

## 4. Conclusion

The histopathological presentation of adenoid (acantholytic) squamous cell carcinoma reflects the prognosis and metastatic behavior of the diseases. The knowledge of histopathological features of ASCC would be a guide to the untrained eye to avoid misdiagnosis and to opt for the best possible treatment protocol to minimize the rate of metastasis or reoccurrence improving the prognosis.

## Figures and Tables

**Table 1:** A brief review of the ADSCC cases of the mandible reported in the literature.

Author	Clinical details	Management	Staging	Follow-up
Chandrakala et al. [5] (2018)	63/F Tobacco chew; ulcerated erythematous mass	Radiotherapy suggested	—	No follow-up

Tsuji et al. [17] (2016)	76/M	—	—	—

Pathak et al. [3] (2014)	55/F; ulceroproliferative mass	Surgery, radiotherapy, and chemotherapy	—	Free of disease (6 months)

Vidyavathi et al. [18] (2012)	40/M Tobacco smoke; polypoidal mass	Surgery	T3 N2 M0	—

Terada [19] (2011)	73/F; exophytic papillary mass	Surgery	—	Free of disease (3 months)

Prasad and Kaur [20] (2010)	70/F; exophytic ulcerated mass	—	—	—

Kusafuka et al. [21] (2006)	64/F	Surgery	cT2cN0cM0	5-month follow-up shows no node/distance metastasis

Driemel et al. [22] (2004)	58/F; exophytic mass	Surgery & radiotherapy	pT4 pN0 cM0 R0	Died after 7 months of initial diagnosis

Jones et al. [23] (1993)	58/M Tobacco smoke	Surgery	—	Free of disease (9 months)

Zaatari and Santoianni [16] (1986)	86/M; nodular growth	Surgery	—	—

## Data Availability

Data is available on request.
